# Segregation dynamics with reinforcement learning and agent based modeling

**DOI:** 10.1038/s41598-020-68447-8

**Published:** 2020-07-16

**Authors:** Egemen Sert, Yaneer Bar-Yam, Alfredo J. Morales

**Affiliations:** 1 New England Complex Systems Institute, Cambridge, MA USA; 2 Department of Electrical and Electronics Engineering, Middle East Technical University, Ankara, Turkey; 3 MIT Media Lab, Cambridge, MA USA

**Keywords:** Computational science, Nonlinear phenomena

## Abstract

Societies are complex. Properties of social systems can be explained by the interplay and weaving of individual actions. Rewards are key to understand people’s choices and decisions. For instance, individual preferences of where to live may lead to the emergence of social segregation. In this paper, we combine Reinforcement Learning (RL) with Agent Based Modeling (ABM) in order to address the self-organizing dynamics of social segregation and explore the space of possibilities that emerge from considering different types of rewards. Our model promotes the creation of interdependencies and interactions among multiple agents of two different kinds that segregate from each other. For this purpose, agents use Deep Q-Networks to make decisions inspired on the rules of the Schelling Segregation model and rewards for interactions. Despite the segregation reward, our experiments show that spatial integration can be achieved by establishing interdependencies among agents of different kinds. They also reveal that segregated areas are more probable to host older people than diverse areas, which attract younger ones. Through this work, we show that the combination of RL and ABM can create an artificial environment for policy makers to observe potential and existing behaviors associated to rules of interactions and rewards.

## Introduction

The recent availability of large datasets collected from various resources, such as digital transactions, location data and government census, is transforming the ways we study and understand social systems^1^. Researchers and policy makers are able to observe and model social interactions and dynamics in great detail, including the structure of friendship networks^2^, the behavior of cities^3^, politically polarized societies^4^, or the spread of information on social media^5^. These studies show the behaviors present in the data but do not explore the space of possibilities that human dynamics may evolve to. Robust policies should consider mechanisms to respond to every type of events^6^, including those that are very rare^7^. Therefore it is crucial to develop simulation environments such that potentially unobserved social dynamics can be assessed empirically.

Agent Based Modeling (ABM) is a generative approach to study natural phenomena based on the interaction of individuals^8^ in social, physical and biological systems^9^. These models show how different types of individual behavior give rise to emergent macroscopic regularities^10,11^ with forecasting capabilities^12^. Applications to social systems include the emergence of wealth distributions^13^, new political actors^14^, multipolarity in interstate systems^15^, and cultural differentiation^16^, among other applications^9^. ABM allows testing core sociological theories against simulations^13^ with emphasis on heterogeneous, autonomous actors with bounded, spatial information^17^. They provide a framework to understand complex behaviors like those of economic systems^18,19^, as well as individual^20^ and organizational^21,22^ decision making processes. These models have been applied for designing distributed systems such as traffic control^23^ and energy management^24^. In biological systems, ABM has shown a remarkable power to explain the spread of diseases^25^, interactions between human body systems^26^, the behavior of ecosystems^27^, and possible links between biological traits and social behaviors^28^.

The Schelling segregation model shows that individual preferences to live away from those that are different may sort social systems in the large scale and generate patterns of social segregation without the need of centralized enforcement^10^. Studies using data show that the model yields segregation over time, regardless of agents’ preferences to live in diverse neighborhoods^29^ or confinement in smaller scales^30^. While integrated societies may be unstable in the long run^31^, another study shows that mixed-race households are more likely to live in integrated neighborhoods than in homogeneous concentrations of either of their parental races or ethnicities^32^. The model has inspired the study of other disciplines that involve the emergence of clusters such as physical systems^33,34^ and cultural groups^35^. While these studies provide deep insight on the underpinning processes of segregation and cases of integration, the inability to experiment with different types of rewards makes it difficult to explore the space of possible behaviors.

Reinforcement Learning (RL) is a simulation method where agents become intelligent and create new, optimal behaviors based on a previously defined structure of rewards and the state of their environment. This method is referred as Multi-Agent Reinforcement Learning (MARL) if multiple agents are employed. Recently, the combination of RL with Deep Learning architectures achieve human level performance in complex tasks, including video gaming^36^, motion in harsh environments^37^, and effective communication networks without assumptions^38^. Moreover, it has been recently applied to study societal issues^39^ such as the emergence of cooperation^40,41^, the Prisoner’s Dilemma^42^ and payoff matrices in equilibrium^43^.

In this paper we extend the standard ABM of social segregation using MARL in order to explore the space of possible behaviors as we modify the structure of rewards and promote the interaction among agents of different kinds. The idea is to observe the behavior of agents that want to segregate from each other when interactions across populations are promoted. We achieve the segregation dynamics by adapting the rules from the Schelling model^10^ in the context of RL. The creation of interdependencies among agents of different kinds is inspired by the dynamics of population models where agents need to interact with each other in order to extend their lifetime^44^. Our experiments show that spatial segregation diminishes as more interdependencies among agents of different kinds are added in the same fashion as if agents are tolerant to one another. Moreover, our results shed light on previously unknown behaviors regarding segregation and the age of individuals which we confirmed using Census data. These methods can be extended to study other type of social phenomena and inform policy makers on possible actions.

The organization of the paper is as follows: In “Methods” we explain the experimental setup including a description of the agents’ behaviors, the structure of rewards and the architecture of the computational framework. “Results” illustrates the experiment outcomes. In “Discussion” we conclude and discuss our results. Future improvements and further methodological details are presented in the Supplement.

**Table 1:** Training parameters of the Deep Q-Networks used during the experiments.

Parameter	Value
Number of episodes	1
Batch size	256
Number of iterations	5,000
Number of training steps	60,000
Experience memory length	1,000,000
Discount factor ($\gamma$)	0.98
Learning rate	0.001
Momentum	0.999
Double network copy parameter ($\tau$)	0.05
Initial exploration rate	0.999
Final exploration rate	0
Exploration decay (per agent action)	100,000

## Methods

We design a model in which two types of agents are simultaneously promoted to both segregate from one another and interact with those of the opposite kind. These behaviors are promoted by providing agents with a set of rewards based on the outcome of their actions. Agents learn over time which actions they should take in order to maximize their rewards. The segregation reward is inspired in the Schelling segregation model where agents decide whether to move further from those that are different from them. Another reward promotes their approach and interaction. By varying the reward of interactions we are able to explore different ways that affect the process of segregation. We achieve the learning process using Deep Q-networks^36^. In this section we explain the state space over which agents are trained and deployed, as well as the set of rewards and rules that determine agents’ behavior.

### The grid world

**Figure 1:**
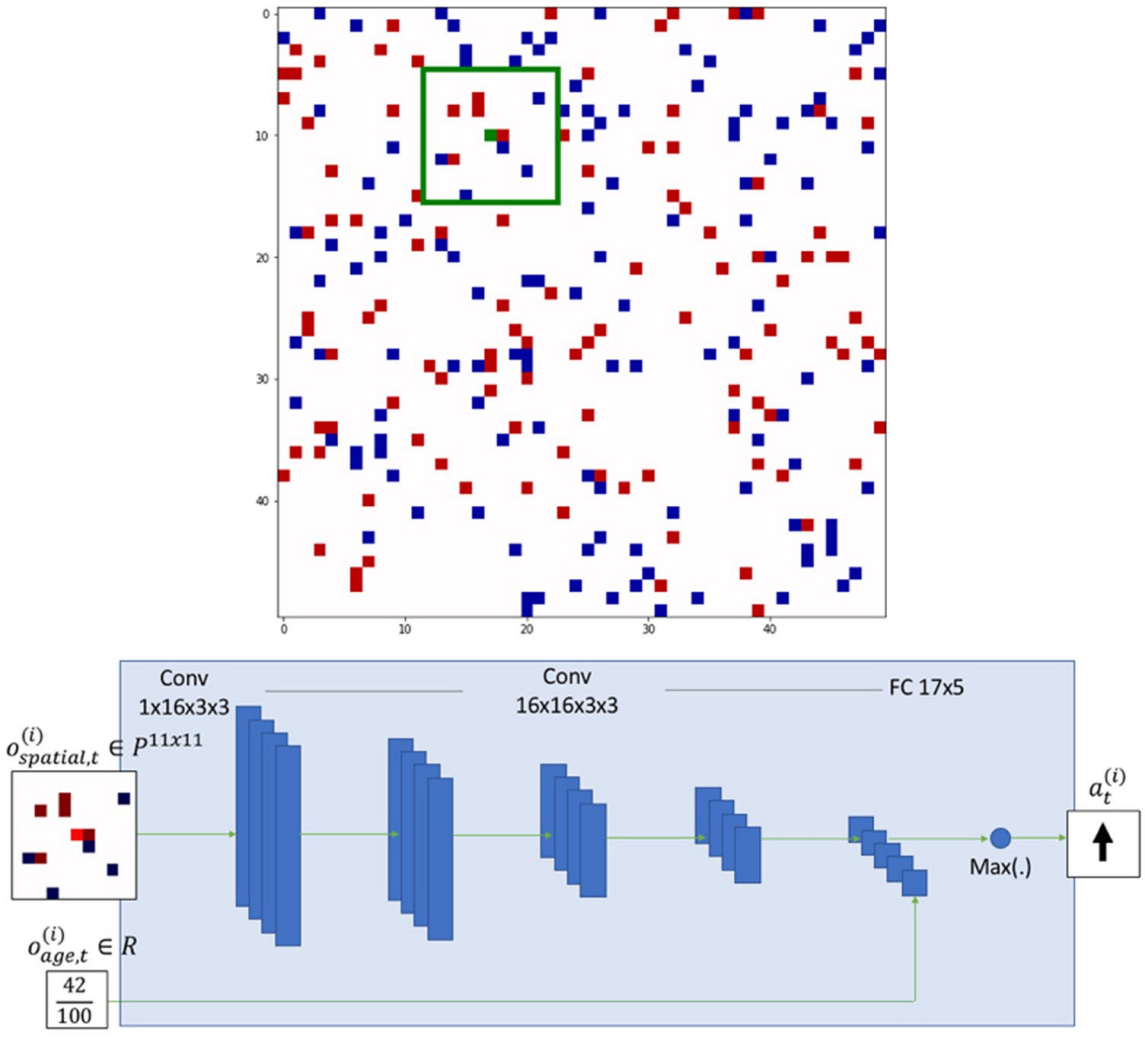
Schematic of the model simulation and network architecture. Top panel: Grid world of experiments. The grid size is 50 $\times$ 50 locations. Red and blue squares denote the two types of agents respectively. White cells represents empty regions. Each type of agent has its own Deep Q-Network. Every agent has a field of view of 11 $\times$ 11 locations. Green border denotes the field of view of the agent illustrated in green. Agents can move across empty spaces. Bottom panel: Example of network structure. Two models are created for $\phi _A$ and $\phi _B$ respectively. Each network receives an input of 11 $\times$ 11 locations, runs it through five convolution steps and concatenates the resulting activations with the agent’s remaining age normalized by the maximum initial age. The feature vector is mapped over the action space using a fully connected layer. The action with the maximum Q-value is taken for the agent.

Our experiments are based on two types of agents, A and B, who live in a 50 $\times$ 50 grid where they can move around and interact with other agents. Figure 1 (top panel) shows an schematic view of the grid world and the agents. Distinct colors (red and blue) indicate the agents’ type. The grid has periodic boundary conditions, meaning that agents that go out one side come back in on the other. Agents observe an 11 $\times$ 11 window of the grid centered around their current location. The green square in Fig. 1 (top panel) represents the observation window of the agent illustrated in green. Agents will evaluate the number of other agents per kind in their observation window in order to decide whether to move and in which direction.

The state of an agent is based on what it sees in its observation window. By taking an action, the agent changes its current state to a new one. Agents evaluate the current state of the observation window and decide which action yields the best rewards. There are five possible actions: to stay still or to move left, right, up or down. This is different from the original Schelling model where agents can move to any location of the grid. All agents take one action at each iteration. The sequence of agents who take actions is chosen randomly.

Agents live for a minimum number of 100 iterations. After an agent dies, a new agent is born in a random location. Agents can extend their lifespan by interacting with agents of the opposite kind. An interaction occurs when an agent moves to a location currently occupied by another agent of the opposite kind. When that happens, we chose a winner and a loser of the interaction. The winner is the one who moves towards the occupied cell and the loser is the one who was at that location. The winner receives a positive reward and extension of its lifespan, and the looser ceases to exist. While this interaction is hostile, it promotes the encounter between agents of different kind. A possible interpretation of the hostile interaction is the emigration of the losing agent out of the neighborhood. As opposed to other implementations of the Schelling model, we create an environment with a low density of agents. It is possible that by having too many agents, the number of possible states that agents can learn from decreases.

### States

States are defined as all the possibilities of an agent observation window. An agent’s observation window consists in a *nxn* patch of the environment where $n = 2*r + 1$ and *r* denotes the radius of the field of view. Each location can take the following values $\{1, 0, -1\}$, where 1 represents agents of similar kind, 0 denotes free locations and -1 represent foes. Observation windows are respectively centered around each agent, whose own location contains a value of 1. We can represent the state space as a string of $n^2$ ternary digits. The agent’s own digit is known to be 1. Therefore, the number of possible strings is $3 ^{n^2 - 1}$. Consequently, in our problem, there are $S(n) = 3 ^{n^2 - 1}$ states available for agents to act upon, where *S*(*n*) denotes the total number of states. The algorithmic complexity grows exponentially with *n* in the order of $O(3^{n^2})$. In our experiments $r=5$ and $n = 11$, yielding more than $10^{57}$ states. On top of this, if each agent can have *M* different age values, the state space is in the order of $O(M~3^{n^2})$. Tabular methods (such as Q-Learning) cannot fit this state space into memory. Therefore, these approaches are not scalable as *n* grows. Function approximation based methods perform better in terms of scalability. Deep Q-networks have a neural network as a function approximator whose domain is the state space and range is the action space, which is more appropriate for this problem. More details about the state space are given in the Supplement (Sect. S3).

### Architecture

Deep Q-networks (DQN)^36^ evaluate actions based on maximizing rewards. Instead of mapping all possible states, we provide agents with a set of rewards that they can use to explore the space of possible states and actions. We create two independent neural networks–one for each type of agent (A and B). An illustration of one of the networks is shown in Fig. 1 (bottom panel). Agents of type A decide from one network and agents of type B decide from another network. The networks are trained as their respective agents take actions and provide them with information. We created two networks in order to have a competitive multi-agent reinforcement learning environment. Otherwise, the environment complexity will be limited by the complexity of a single network. By adding a new network we increase the complexity of the model with the new network and the interaction of both networks.

Mathematically, agents of type A are represented as $-1$, B as $+1$, and empty spaces as 0 on the grid. Each agent’s field of view is normalized by its type such that friends are represented as $+1$ and foes as $-1$. Hence every agent’s spatial observation at time *t* is $O_{spatial, t}^{(i)} \in P^{11x11} ~ | ~ P \in \{-1, 0, 1\}$. Moreover, every agent has the information of its remaining normalized life time, represented as $O_{age, t}^{(i)} \in R$. Full observation of the agent *i* at time *t* is $o_t^{i} \in O_t^{(i)} = O_{spatial,t}^{(i)} \cup O_{age,t}^{(i)}$. Let $\phi _A$ and $\phi _B$ denote the Q-Networks of type A and B agents. Then the networks’ goal is to satisfy Eqs. 1 and 2.

1 $$\begin{aligned} \phi _A^*= & {} \arg \max _{\phi _A} {\mathbb {E}} \left[ \sum _{t=0}^{T}\sum _{i=1}^{N_A} \gamma ^{t}r_t^{(i)} | o_t^{(i)}\right] \end{aligned}$$

2 $$\begin{aligned} \phi _B^*= & {} \arg \max _{\phi _B} {\mathbb {E}} \left[ \sum _{t=0}^{T}\sum _{i=1}^{N_B} \gamma ^{t}r_t^{(i)} | o_t^{(i)}\right] \end{aligned}$$

where $N_X$ denotes the number of agents of type *X*, $\gamma$ denotes the discount factor, $r_t$ denotes the reward at time *t* and $Q_{\phi _{X}}(.)$ denotes the Q-Network of agents of type *X*.

Each network is initialized with the same parameters. In order to homogenize the networks’ inputs, we normalize the observation windows by the agents’ own kind, such that positive and negative values respectively represent equal and opposite kind for each agent. Actions are taken by following $\epsilon$-Greedy exploration strategy. This strategy is used for improving the learning process of the state space, especially during the first stages. If we do not use it, the learning process may not converge, because some critical states may not be explored. It consists in taking a random action instead of the recommended one by the neural network with a probability $\epsilon$ that decays exponentially over time. In order to avoid over-fitting of parameters and approximate the rewards appropriately, we need to stabilize the learning process. We use the algorithm Adam optimizer^45^ to efficiently update the network parameters and minimize approximation errors at each iteration. Experience Replay^46^ is applied for mitigating time correlation among the inputs of the neural network. Otherwise, DQN may overfit the current state and its variants. Double Q-Learning^47^ is used such that very noisy learning signal would not diverge the learning process. If Double Q-Learning is not used, an outlier batch of samples might skew the parameters away from minima.

We run one episode per experiment. Each episode is comprised of 5,000 iterations. Each experiment is repeated 10 times for statistical analysis. Networks’ details are given in Fig. 1 (bottom) and training details are given in Table 1. As a reference, in terms of performance, one iteration takes roughly 0.38 seconds on a 3.1 GHz Intel Core i5 processor and 8 GB 2133MHz LPDDR3 memory.

### Rewards

The model rewards, *R*, are scalar values that we respectively provide to agents at each interaction after evaluating their current state and action. This scalar results from the sum of a set of specific rewards: $R=SR+IR+VR+DR+OR+TR$, that we explain in this section. The rewards are as follow:

- **Segregation reward (SR)**. This reward promotes agents’ segregation, in the form: $SR=s-\alpha d$, where *s* is the number of agents of similar kind within the agent’s observation window, *d* is the number of agents of different kind within the observation window and $\alpha \in [0,1]$ is a parameter we use to control the intolerance of agents to be next to those that are different from them. The segregation parameter $\alpha$ is analogous to the threshold used in the original Schelling model. In the Supplement (Sect. S2) we present the mathematical relationship of this reward with the intolerance threshold from the Schelling model.
- **Interdependence reward (IR)**. This reward promotes interactions among agents of different kind. When an agent meets another agent of different kind, we choose a winner and a loser of the interaction. The winner is the one who moves to the cell occupied by the other agent. The winner receives a positive reward and an extension of its lifetime by one iteration. The loser ceases to exist. We use the IR as a parameter we can vary $IR \in [0,100]$ in order to promote interactions among agents of different kind.
- **Vigilance reward (VR)**. This reward promotes agents to stay alive by providing $VR=0.1$ reward for every time step they survive and $VR=0$ when they die. We include this reward such that agents learn during the early stages that they need to stay alive in order to collect more rewards. Larger values of VR may override other rewards leading agents to just stay alive without exploring other behaviors.
- **Death reward (DR)**. We punish agents who die or lose interactions against agents of the opposite kind. Agents receive $DR=-1$ reward when they die or $DR=0$ when they stay alive. Agents must learn that dying is bad. Otherwise dying would not have an effect on the total rewards collected by agents and it would be more difficult for them to avoid risky situations and reach older ages.
- **Occlusion reward (OR)**. This reward punishes movements towards occupied cells between agents of the same kind. If an agent tries to move towards an area occupied by an agent of its own kind, the agent receives $OC=-1$ reward. If the agent moves towards a free cell, it receives $OC=0$. In order for agents to understand that they cannot try to move to an occupied cell but to move towards free ones, we need to explicitly reward negatively those actions. Otherwise it could be the case that some agents try to move to occupy cells and waste an action that could have been used to explore the remaining space.
- **Stillness reward (TR)**. This reward promotes the exploration of space by punishing staying still. Agents who choose to stay still receive $TR=-1$ reward. Agents who chose to move receive $TR=0$. If we do not punish staying still, some agents may chose to do so and the space would not be sufficiently explored. Staying still could be a local minimum in the function approximated by the neural network and agents could believe that it is the best action.

## Results

Experiments are conducted by setting up different values of rewards and observing the emergent collective behavior associated with each experiment. During simulations, agents explore the space of possible behaviors and inform which behaviors are promoted under certain rewards and environmental rules. As a result, we create an artificial environment for testing hypotheses and obtaining information through simulations hard to anticipate given the complexity of the space of possibilities.

### Modeling segregation

**Figure 2:**
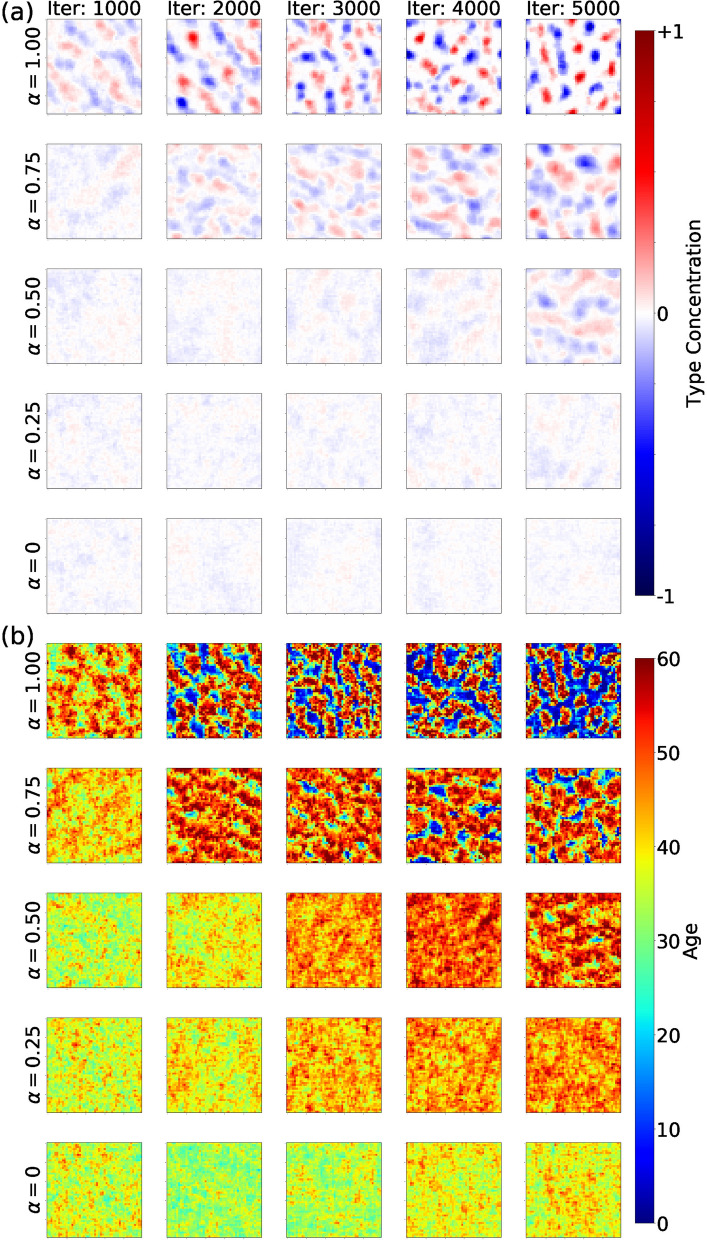
Agents collective behavior for multiple values of segregation reward $\alpha$ (rows) at multiple times (columns). Rows represent outcomes associated to different values of segregation reward ($\alpha$). Columns show the state of the system at different points of the simulation. In Panel (**a**) colors indicate the concentration of both types of agents (blue and red). White indicates the average pattern. In Panel (**b**) color indicates the age of agents irrespective of their type. Scales in figure.

We reward agents to segregate from those of different kind using a parameter $\alpha$, which represents the intolerance to be next to those that are different (see “Methods”). The segregation parameter ranges between $\alpha =0$ in the case of maximum tolerance to the other population and $\alpha =1$ in the case of maximum intolerance. Figure 2 shows the emergent collective behavior for multiple values of $\alpha$ (rows) at multiple times of the simulation (columns). Rows represent outcomes associated to different values of the segregation parameter ($\alpha$). Columns show the state of the system at different points of the simulation. Experiments are initialized with equal initial conditions and random seed. The heat maps are obtained by averaging over the last 1,000 iterations. We share videos of segregation experiments at the following links: ($\alpha =0$) https://youtu.be/1qfbg4NLp8w, ($\alpha =0.25$) https://youtu.be/8nqll-jh9Ds, ($\alpha =0.50$) https://youtu.be/LXAKN3GrzEo, ($\alpha =0.75$) https://youtu.be/doNt7UJBqbg, ($\alpha =1.00$) https://youtu.be/YP0FGUo4tH4.

In Panel (a) we show the average type occupation per location. Red regions denote biased occupation of type A agents and blue regions denote biased occupation of type B agents. White areas indicate the average pattern. Lower values of $\alpha$ yield mostly white spaces, indicating a mixed population. As we increase $\alpha$ the segregation of agents begins. With high levels of $\alpha$ the segregation is pronounced and blue and red segregated clusters emerge. This happens even within the first 1,000 iteration where the model could still be still learning. However, similarly to the original Schelling segregation model, segregation still occurs for smaller values of $\alpha$ in the long run (see $\alpha =0.5$).

The white regions for lower values of $\alpha$ indicate mixing, while the white regions of higher values of alpha are characterized for being emptier. In Panel (b) we color locations by the age of agents irrespective of their type. The agent age increases as color shades from blue to red. In the bottom row ($\alpha =0$) the mixing of types and ages is high with respect to $\alpha =1$. As we increase the reward for segregation, clusters of specific types of agents arise and their age distribution is characterized for being heterogeneous. The white inter-cluster regions show a very low average age. The segregated clusters host older agents inside and younger ones in the periphery (see $\alpha =1$). This shows that with higher rewards for segregation, the population has very little interaction across type or even clusters of their same kind. Most agents remain mostly near their cluster.

**Figure 3:**
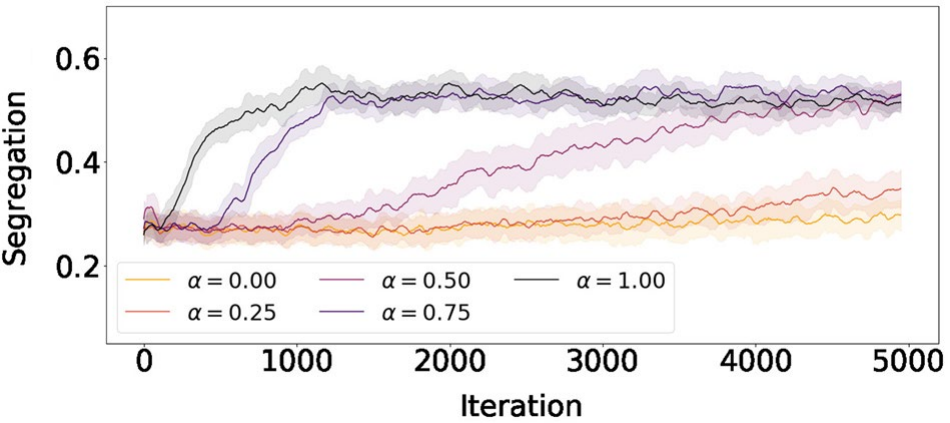
Segregation dynamics for multiple values of segregation reward ($\alpha$). Colors correspond to the results for multiple values of segregation reward ($\alpha$), ranging from yellow (low) to black (high). The curves are obtained by averaging 50 iterations over 10 experiment realizations. Shades denote the standard deviation across experiments.

We measure segregation among agents using multiscale entropy. We slide windows of three different sizes (6 $\times$ 6, 12 $\times$ 12 and 25 $\times$ 25) over the whole grid. For each window, we count the number agents per type, normalize their counts to probabilities and calculate the entropy of the distributions. At each iteration, we calculate the average entropy, $\langle e\rangle$, across all windows and scales. The resulting segregation has the form $1-\langle e\rangle$. See Sect. S4 for more details on the multiscale entropy calculation. In Fig. 3 we present the dynamics of segregation for multiple values of $\alpha$ (color). High values of $\alpha$ yield segregated spaces very fast. Intermediate values of $\alpha$ get segregated but take longer to reach the same level of segregation. Lower values of $\alpha$ remain mixed for a much longer time. Unlike the classic Schelling model, RL agents are constantly rewarded for their actions. Therefore, instead of reaching an equilibrium where everyone is happy, they continue to move and learn from their environment. This reinforcing dynamics can lead to segregation for smaller values of $\alpha$ at a very slow pace.

### Modeling interdependencies

We provide rewards to create interactions and interdependencies among both populations. For this purpose, we combine the segregation dynamics with the interdependence reward (IR). The interdependence reward is given when agents of different kinds interact with one another (see “Methods” for more details). Interactions occur when an agent of the opposite kind attempts to move to an occupied location. The one who moves towards the occupied location gets a positive reward and life-extension. The one who was in the occupied location dies and gets a negative death reward.

Although hostile, this interaction may reward positively agents. Therefore, we use it to promote interactions and create interdependencies among both populations.

**Figure 4:**
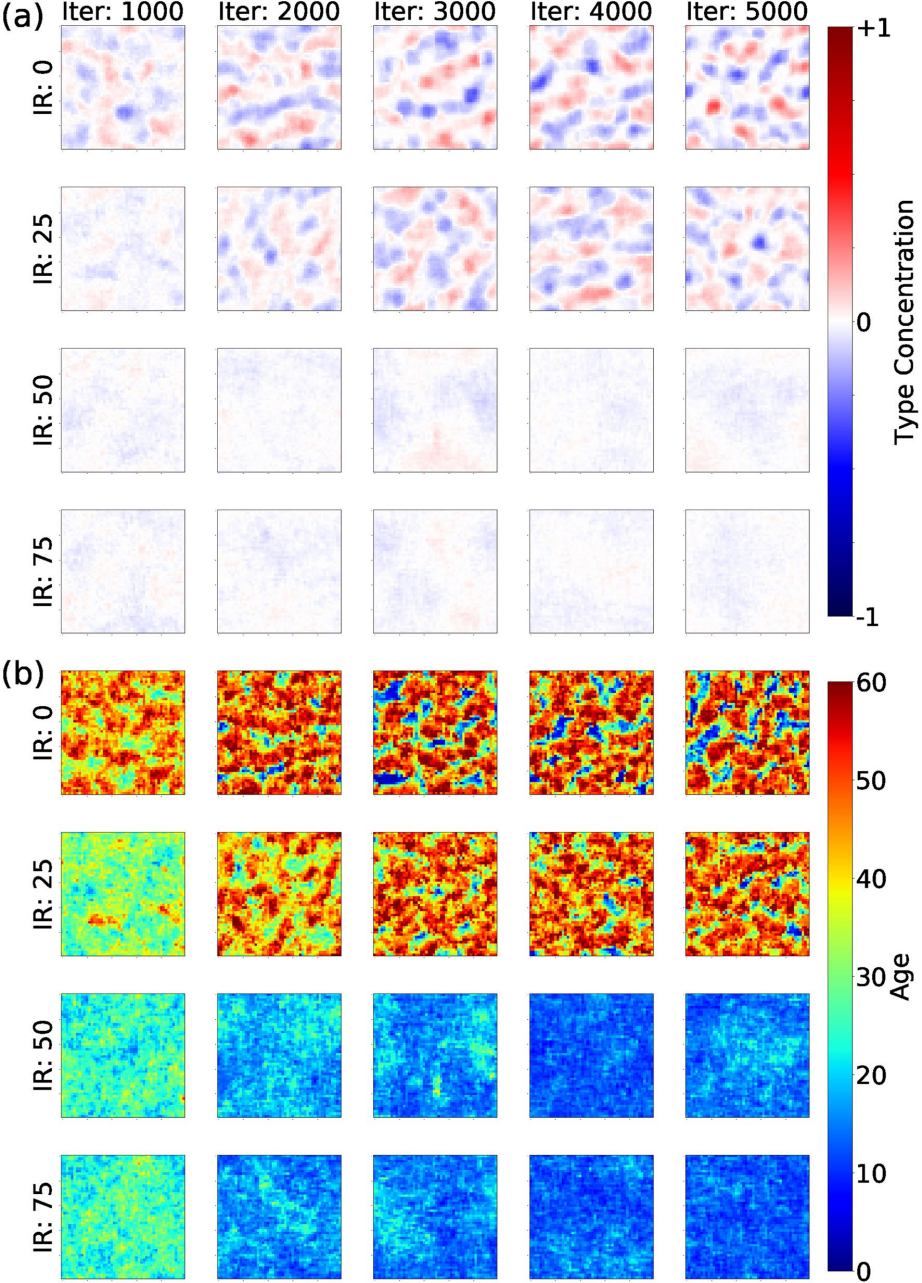
Agents collective behavior for multiple values of interdependence reward (IR) at multiple times (columns) for maximum segregation parameter ($\alpha =1$). Rows represent outcomes associated to different values of interdependence reward (IR). Columns show the state of the system at different points of the simulation. In Panel (**a**) colors indicate the concentration of both types of agents (blue and red). White indicates the average pattern. In Panel (**b**) color indicates the age of agents irrespective of their type. Scales in figure.

Interdependence rewards diminish spatial segregation among different types. In Fig. 4a we show the collective behavior of the population after setting the maximum segregation parameter ($\alpha =1$) and varying the values of interdependence reward. We use heat maps proportional to the probability of agents location during simulations according to their type (in a similar fashion as in Fig. 2). Experiments are initialized with equal initial conditions and random seed. The heat maps are obtained by averaging over the last 1,000 iterations and visualized over one trial of the experiments. Red and blue regions show biased occupation of agents A and B respectively. White areas indicate the average pattern. Without rewarding for interdependencies (IR = 0), the dynamics of segregation quickly result in patches of segregated groups (top row). As interdependence rewards increase, the probability of locations being occupied by agents of type A or B becomes uniform and plots become white (bottom right panels). By creating interdependencies among them, agents increase their interactions and reduce the spatial segregation. Videos of interdependence experiments can be found at the following links: (IR: 0) https://youtu.be/YP0FGUo4tH4, (IR: 25) https://youtu.be/2dxP-aJdM4A, (IR: 50) https://youtu.be/cO4Jh75qYiQ, (IR: 75) https://youtu.be/EuWE1ydhdHo.

**Figure 5:**
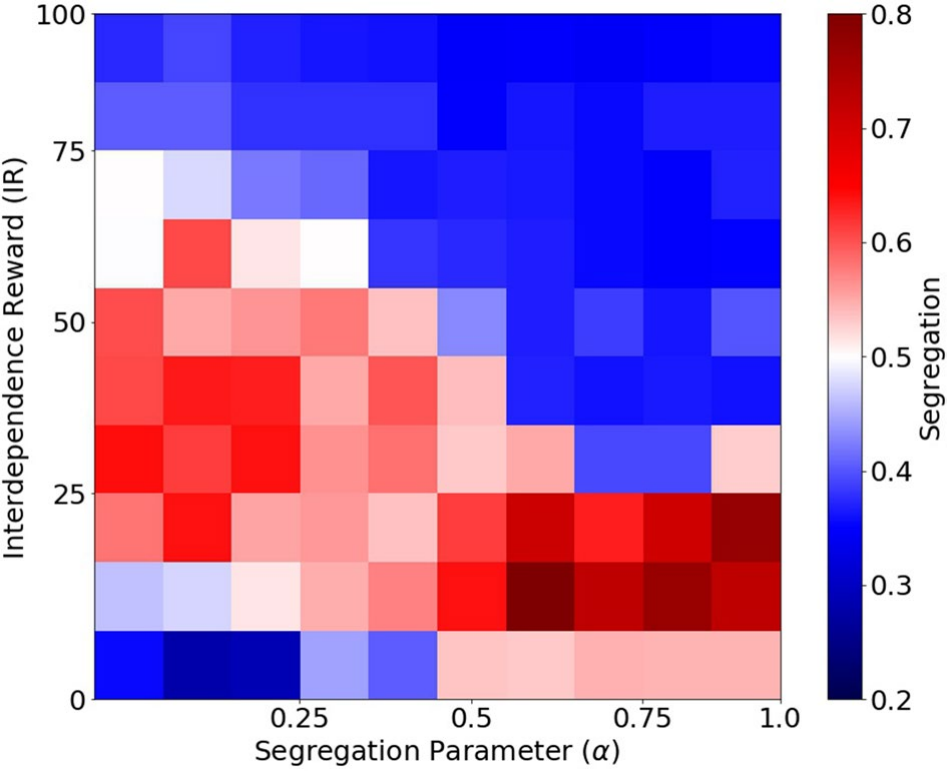
Segregation values for multiple values of segregation parameter ($\alpha$) and interdependence reward (IR). Colors correspond to amount of segregation measured in the last 1,000 iterations of the simulation. Scale in figure.

We explore multiple combinations of the segregation parameter $\alpha$ and the interdependence reward (IR). The resulting segregation of those simulations is visualized in Fig. 5. The x-axis represents the segregation parameter $\alpha$ and the y-axis represents the interdependence reward (IR). The figure shows a contour plot of the expected amount of segregation in the system during the last 1,000 iterations. We calculate segregation using entropy as in Fig. 3. Red regions indicate high segregation and blue regions show lower segregation. Segregation is high (red) when promoted (high $\alpha$) and interdependencies are not rewarded. As interdependencies increase, the agents mix and the spatial segregation is significantly reduced (blue), even for high values of $\alpha$. Therefore, high levels of interdependencies seem to counter the rewards for segregation. The resulting mixing for high levels of interdependencies are comparable to very low levels of $\alpha$.

### Age dynamics

Age is one of the parameters we input the DQN with in order to recommend actions. We analyze the effects of age in the both the emergent behaviors of agents, as well as biases in the actions they take.

**Figure 6:**
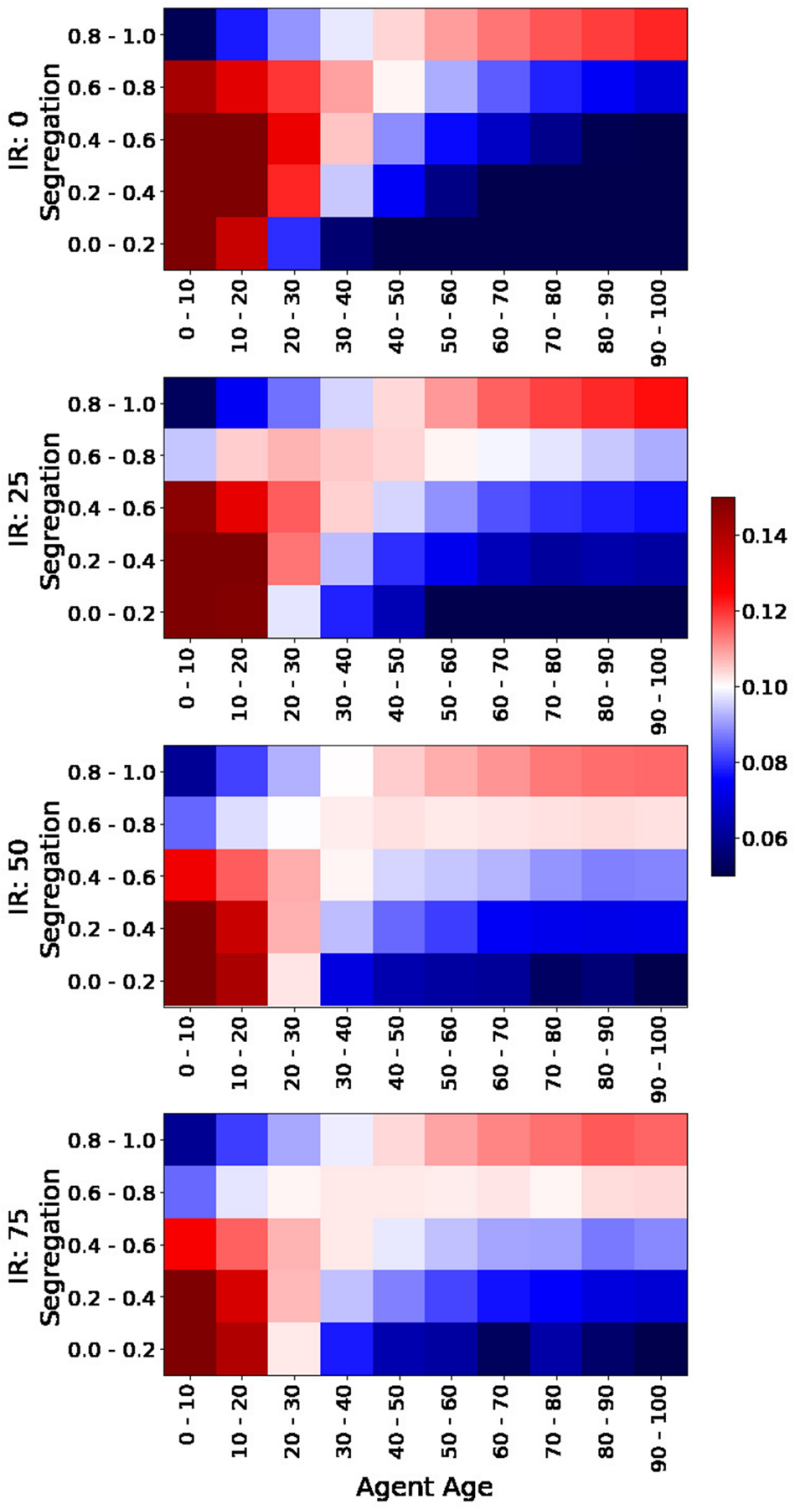
Probability distribution of age groups conditional on segregation of observation windows. Each panel shows the probabilities of finding agents at each age group (columns) at different levels of segregation in their observation windows (rows) during the last 1,000 iterations. There is one panel per each value of interdependence reward (IR). The segregation parameter $\alpha =1$ for all panels. The plot shows the average of 10 experiment replicas. Scale in figure.

We first studied the probability distributions of age groups conditional on the segregation of their observation windows during the last 1,000 iterations. For this purpose, we split the population in ten age groups and measure the relative number of agents of similar kind within their observation windows. We split this measure of segregation in 5 bins and count the number of agents at each age group and segregation bin. In order to avoid imbalanced samples, we first normalize by the number of agents per age group and later by the segregation bin. The results are presented in Fig. 6 for multiple values of IR (and setting $\alpha =1$). Red squares indicate a higher probability of finding a given age group at a given level of segregation, while blue squares indicate lower probabilities. The figure shows that older agents have significantly more segregated observation windows than younger agents who live in more diverse areas. This effect is naturally more pronounced for lower values of IR and less pronounced as we increase IR. However, the observation that older agents prefer to be segregated remains consistent. In the Supplement, we present analogous plots for multiple values of the segregation parameter $\alpha$ and population types (see Sect. S5).

**Figure 7:**
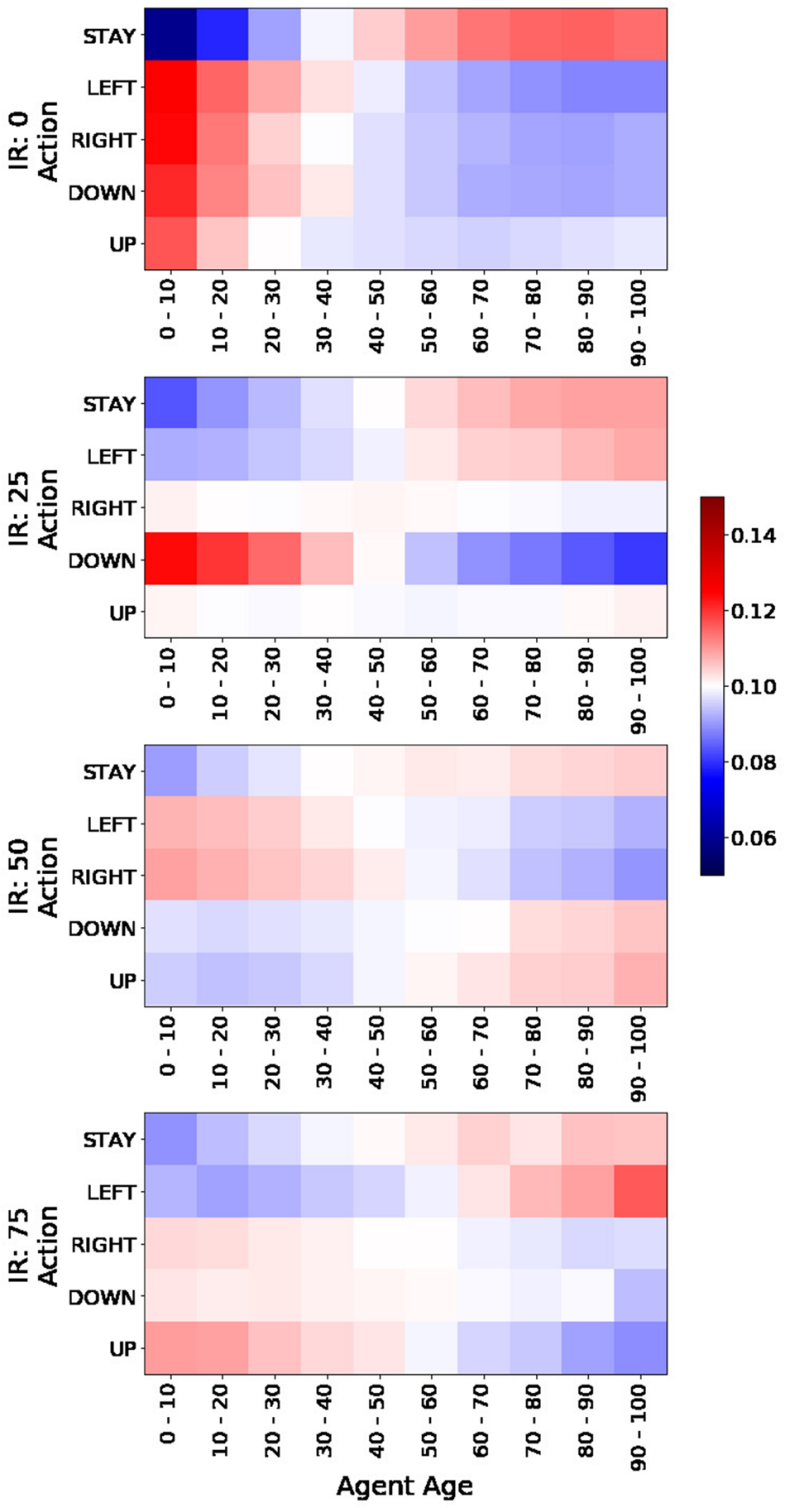
Probability distribution of age groups conditional on actions. Each panel shows the probabilities of finding agents at each age group (columns) for each of the possible actions (rows) during the last 1,000 iterations. There is one panel per each value of interdependence reward (IR). The segregation parameter $\alpha =1$ for all panels. The plot shows the average of 10 experiment replicas. Scale in figure.

We also study biases in the actions taken by agents according to their age group. We analyze the probability of age groups conditional on the actions taken during the last 1,000 iterations similarly to Fig. 6. The results are presented in Fig. 7 for multiple values or IR (and setting $\alpha =1$). Red squares indicate higher probabilities of agents taking different actions according to their age group and blue squares represent lower densities. The figure shows that older agents tend to stay more still than younger agents who seem to explore the space further. It also shows that certain movements are biased towards certain age groups and that stay probabilities become smoother as we increase IR. Similar plots as a function of $\alpha$ and population types are presented in the Supplement (see Sect. S6). The behavior shown in Fig. 7 is consistent across both types of agents (see Fig. S8).

People are older in segregated areas. The model shows that older agents are more segregated than younger ones. We believe that in our simulations older agents become more segregated because the expected rewards for other social interactions are lower than staying safe. This behavior has been verified with human behavior using Census data. We analyzed the relationship between age and segregation using Census data across the whole US (see Sect. S5). A segregation metric based on racial entropy correlated positively with median age by census tract (r = 0.4). Our simulation shed light on an observation that is not trivial about current societies.

## Discussion

We created an artificial environment for testing rules of interactions and rewards by observing the behaviors that emerge when applied to multi-agent populations. Rewards can generate surprising behaviors because of the complexity of social systems. As problems become complex, evolutionary computing is necessary to achieve sustainable solutions. We combine agent based modeling (ABM) with artificial intelligence (RL) in order to explore the space of solutions associated to promoted rewards. RL provides ABM the information processing capabilities that enables the exploration of strategies that satisfy the conditions imposed by the interaction rules. In turn, ABM provide RL with access to models of collective behavior that achieve emergence and complexity. While ABMs provide access to the complexity of the problem space, RL facilitates the exploration of the solution space. Our methodology opens a new avenue for policy makers to design and test incentives in artificial environments.

## Supplementary information

Supplementary information

## Data Availability

The source code of the model implementation is available at: https://github.com/egemensert/segregation. See Sect. S7 for more detailed explanation.
