# Supplementary material for: Segregation dynamics with reinforcement learning and agent based modeling
Source: Sci Rep. 2020 Jul 16;10:11771. doi: 10.1038/s41598-020-68447-8 (PMC7367354; doi:10.1038/s41598-020-68447-8)
Supplement: Supplementary file 1 — Supplementary information [file 41598_2020_68447_MOESM1_ESM.pdf]

Supplement: Segregation Dynamics with Reinforcement Learning and Agent Based Modeling

Egemen Sert[1,2], Yaneer Bar-Yam[1] and Alfredo J. Morales[1,3,*]

[1]New England Complex Systems Institute, Cambridge, MA. [2]Department of Electrical and Electronics Engineering, Middle East Technical University, Ankara, Turkey. [3]MIT Media Lab, Cambridge, MA.. [*]Corresponding author: alfredom@mit.edu

ABSTRACT

Societies are complex. Properties of social systems can be explained by the interplay and weaving of individual actions. Rewards are key to understand people's choices and decisions. For instance, individual preferences of where to live may lead to the emergence of social segregation. In this paper, we combine Reinforcement Learning (RL) with Agent Based Modeling (ABM) in order to address the self-organizing dynamics of social segregation and explore the space of possibilities that emerge from considering different types of rewards. Our model promotes the creation of interdependencies and interactions among multiple agents of two different kinds that segregate from each other. For this purpose, agents use Deep Q-Networks to make decisions inspired on the rules of the Schelling Segregation model and rewards for interactions. Despite the segregation reward, our experiments show that spatial integration can be achieved by establishing interdependencies among agents of different kinds. They also reveal that segregated areas are more probable to host older people than diverse areas, which attract younger ones. Through this work, we show that the combination of RL and ABM can create an artificial environment for policy makers to observe potential and existing behaviors associated to rules of interactions and rewards.

S1 Future Work

There are many potential improvements to our work. We classify directions of future work under three categories: representation, training and experimentation. Our method can be advanced by representing agents more realistically such as introducing heterogeneous personalities to agents or facilitating network structure over agents to promote alliances. Moreover, training RL agents yield better results with sophisticated exploration strategies[1–3]. In addition to exploration strategies, MARL is shown to perform better with curriculum learning[4]. Our aim is to extend the work on multi agent curriculum learning to our problem.

We are currently working on extending this artificial environment to other ABMs, i.e. Axelrod model[5]. Our goal is to develop an easy interface where policy makers and AI researchers can collaborate on solving societal problems.

S2 Relation Between Schelling Segregation Model and the Segregation Reward

In Schelling Segregation Model[6], agents move randomly if the the relative number of agents of the opposite kind (d) with respect to the total number of agents in their surrounding ($s+d$) exceeds a threshold γ (see Equation 1). This relation is equivalent to the Equation 2 when $0 \leq \gamma < 1$. If $\alpha = \frac{1-\gamma}{\gamma}$ we get the segregation reward (see Equation 3).

$$\frac{d}{s+d} \geq \gamma \tag{1}$$

$$s - \frac{1-\gamma}{\gamma} d \leq 0 \tag{2}$$

$$s - \alpha d \leq 0 \tag{3}$$

Note that although the reward functions are equivalent in Schelling Segregation Model and in our segregation experiments, stopping criteria are different. In Schelling Segregation Model, agents halt whenever their segregation exceeds the threshold. In our model, agents satisfying a threshold is only a baseline where the reward becomes positive. Furthermore, in our model, agents try to maximize their reward, in other words, regardless of α, agents prefer full segregation over satisfying the threshold. Hence, although the rewards are, simulations are not equivalent.

S3 State Space and RL Model Selection

In this paper, we aim to create an environment on modeling social phenomena by using ABMs with Reinforcement Learning. Note that there are many social phenomena along with many type of ABMs and Reinforcement Learning models. As our setup is nonlinear and time variant, we needed to employ a complex modeling approach.

There exists many modeling approaches. In this research, we employ numerical methods. Among various numerical methods, we have selected Deep Q Learning based on memory concerns. When only spatial state is taken into account,state space complexity (memory requirement) of our problem is equivalent to number of distinct $n^2 - 1$ length strings that can be written with an alphabet of three characters. Moreover, if we assume each agent can have M different age value, state space becomes $M3^{n^2-1}$. In the following paragraph, we will show the equivalence.

In our 50x50 grid space, agent kinds are represented either by -1 or $+1$ where -1 denotes opposing kind and $+1$ denotes the same kind with the agent. Empty spaces are represented by number 0. Consequently, any point on 50x50 grid space can take one of the three values $\{-1,0,1\}$. According to our setup, agents live on grid space. Each agent has a field of view (FOV) having a fixed radius r. Then, one side of agent's FOV has $n = 2r+1$ pixels, r pixels to the left and right of the agent and the agent centered in the middle. Hence, an agent's FOV contains n^2 pixels where the center pixel is the agent (always equal to $+1$). Making a state consist of $n^2 - 1$ pixels where each pixel can take one of the three values $\{-1,0,1\}$. Therefore, number of distinct states given n per network is $S(n) = 3^{n^2-1}$ when only the spatial state is investigated (see Figure S1). If each agent can have M different age values, the total state space is $S(n) = M3^{n^2-1}$

Large state space is a common problem in Reinforcement Learning. To overcome it, numerous solutions exist. First of all, symmetry conditions can be exploited to reduce the state space (where mirror inverse or 180 deg rotations are collapsed to one state). Aside from this, efficient featurization methods such as Tile Coding can be employed. We refer interested readers to Sutton and Barto's Reinforcement Learning book Section II.9.5[1] (the book will be referred as the RL book). It is important to note that although hand designed features can be very efficient, it is highly problem specific. What we aim to achieve is developing a system optimized to explore space of possibilities, given a policy. In order for researchers to not spend much time finding an efficient feature representation, we have used Deep Q Learning as our model. Where feature extraction process is jointly learned with policy using a differentiable nonlinear function. As this approach is approximate and learnable by reward signals on and off-policy (please see Section I.6, II.9, II.10 and II.11 of the RL book) it can be generalized to many problems with relative ease[7].

It is important to note that, although our approach is scalable, the action space of the optimizer is discrete. For continuous action space optimizers, we refer researchers to Policy Gradient or Actor-Critic based methods (see Section II.13 of the RL book) and their deep learning variants[8–11].

S4 Measuring Segregation

We have measured segregation using multiscale entropy. Entropy of the grid space on $n-$Scale is as following: We look at nxn patches of the grid, calculate entropy on each patch and average patch entropies over the grid space[2]. For example, for $5-$Scale entropy, entropy values of a hundred 5x5 patches are averaged. While calculating entropy of a patch, empty spaces are not count. If there are 4 A type agents and 5 B type agents at an nxn patch, the entropy E_n is calculated as $E_n = -\left(\frac{4}{9}\log_2\frac{4}{9} + \frac{5}{9}\log_2\frac{5}{9}\right)$ (see Equation 4). In Figure S2 we show how entropy values change across different scales for varying iterations of an experiment with $\alpha = 1$ and $IR = 0$. In our experiments, multiscale entropy is calculated by averaging 6x6, 12x12 and 25x25 scale entropy values (shown in dashed gray lines in Figure S2). Entropy is maximum when number of agents per kind is equal in each patch (fully integrated) and minimum when only one kind of agent exists in each patch (fully segregated).

Note that segregation values are not zero at the first iteration. This nonzero value is related to the distribution of agent kinds and voids on the grid space. A grid space is initialized such that at each grid location a three sided dice (empty space, type A, type B) is thrown where probability of empty space is $p_v = 0.9$, probability of agent A is $p_A = 0.05$, and probability of agent B is $p_B = 0.05$. Therefore, there is a probability associated to having A number of one type and B number of another type in N pixels drawn from a multinomial distribution, as shown in Equation 5. Note that $A+B \leq N$ and $1 \leq A \leq p_A N$ and $B \leq p_B N$. Finally, expected entropy in N pixels is given by Equation 6. Therefore, by solving the equation 6 for $N = 6^2$, $N = 12^2$ and $N = 25^2$ and averaging over three scales, baseline multiscale entropy and segregation values can be found. He have calculated the baseline segregation as 0.21[3].

---

[1] http://incompleteideas.net/book/the-book-2nd.html

[2] In the bounded space setting, if overflow from grid space occurs while calculating entropy of a patch, overflowing locations are filled with empty space. In unbounded setting, overflows are filled with corresponding grid locations.

[3] Please visit https://colab.research.google.com/drive/1zWOURa_iyph6YgIhC-ntiKZ7JrwDm0vT for baseline calculation

$$\varepsilon(A,B) = -E\left[\frac{A}{A+B}\log_2\frac{A}{A+B} + \frac{B}{A+B}\log_2\frac{B}{A+B}\right] \tag{4}$$

$$p(A,B,N) = \frac{N!}{A!B!(N-(A+B))!} {p_A}^A * {p_B}^B * {p_V}^N \tag{5}$$

$$E[\varepsilon] = \sum_{(a,b)\ s.t\ 1\leq A+B\leq N} p(a,b,N)\varepsilon(a,b) \qquad 1 \leq A \leq p_A N \qquad 0 \leq B \leq p_B N \tag{6}$$

S5 Age and Segregation

We studied the probability distributions of age groups conditional on the segregation of their observation windows during the last 1000 iterations for multiple values of the segregation parameter α. We split the population in ten age groups and measure the relative number of agents of similar kind within their observation windows. We split this measure of segregation in 5 bins and count the number of agents at each age group and segregation bin. In order to avoid imbalanced samples we first normalize by the number of agents per age group and later by the segregation bin. The results are presented in Figure S3 for multiple values of the segregation parameter α and IR=0. Red squares indicate a higher probability of finding a given age group at a given level of segregation, while blue squares indicate lower probabilities. The figure shows that older agents have significantly more segregated observation windows than younger agents who live in more diverse areas. This effect is naturally more pronounced for higher values of α and less pronounced as we decrease α. Figures S4 and S5 show analogous plots for the different types of population.

The behavior has been observed in the model and verified it with human behavior using Census data. We analyzed the relationship between age and segregation using Census data across the whole US. A segregation metric based on racial entropy correlated positively with median age by census tract (r=0.4). In Figure S6 we present a scatter plot of the segregation metric (x-axis) and average age (y-axis) of each census tract (dots). The segregation metric is the compliment of the entropy of the distribution of races per census tract.

S6 Agent Bias

We study biases in the actions taken by agents according to their age group and agent type. We analyze the probability of age groups conditional on the actions taken during the last 1000 iterations. The results are presented for multiple values or the segregation parameter α in Figure S7 and for multiple values of IR in Figure S8. We split the population by type. In both figures, left column shows the results of agents A and right column shows the results of agents B. Red squares indicate higher probabilities of agents taking different actions according to their age group and blue squares represent lower densities. Figure S7 shows that agents have very consistent behaviors in terms of actions as a function of the segregation parameter α. Figure S8 shows agents have different types of behavior in terms of IR. Agents B (right column) seem more erratic than Agents A (left column) which seem more coherent.

S7 Experimental Setup

In our implementation, there are three main classes: Agent, Mind, and Environment. The Agent class holds the agent's kind, location on the grid and field of view (FOV). Mind class contains the Deep Q-networks (DQN) which are implemented using PyTorch library. Their purpose is to collect agent states and action pairs. They learn a policy to maximize rewards. Finally, Environment class holds the grid space with references of the agents and their respective Mind (DQN). Experiments are iterated via simulation engine implemented within the Environment class. The engine is responsible for executing functions regarding how simulation would evolve when agents take action e.g. staying still, interacting with the same kind or opponent, moving spatially and so on. These functions take an agent class as input and returns a reward for the given action. Therefore, by carefully implementing action functions, various simulation environments can be constructed.

Engine of the environment is as following. A set of actions are taken per iteration until the final iteration is reached:

- Shuffle agents' action taking order.
- For each Agent in agents' action taking order.
  - Get Agent's field of view (FOV) and age.

  - Sample action from the agent respective Mind by supplying the agent FOV and age/100. The FOV and age/100 will be referred as agent's state.
  - Depending on Agent's action, invoke respective function: on opponent, on same, etc., and receive the reward.
  - Register agent's state action and reward tuple to each Mind.

- With some probability, re-spawn dead agents at a random location.
- Train each Mind by randomly sampling BATCH SIZE of (state, action, reward) tuples from agents' memory (one per kind).

References

1. Nikolov, N., Kirschner, J., Berkenkamp, F. & Krause, A. Information-directed exploration for deep reinforcement learning. *arXiv preprint arXiv:1812.07544* (2018).
2. Tang, H. *et al.* # exploration: A study of count-based exploration for deep reinforcement learning. In *Advances in Neural Information Processing Systems*, 2753–2762 (2017).
3. Fu, J., Co-Reyes, J. & Levine, S. Ex2: Exploration with exemplar models for deep reinforcement learning. In *Advances in Neural Information Processing Systems*, 2577–2587 (2017).
4. Bansal, T., Pachocki, J., Sidor, S., Sutskever, I. & Mordatch, I. Emergent complexity via multi-agent competition. *arXiv preprint arXiv:1710.03748* (2017).
5. Axelrod, R. The dissemination of culture: A model with local convergence and global polarization. *J. conflict resolution* **41**, 203–226 (1997).
6. Schelling, T. C. Dynamic models of segregation. *J. mathematical sociology* **1**, 143–186 (1971).
7. Mnih, V. *et al.* Human-level control through deep reinforcement learning. *Nature* **518**, 529 (2015).
8. Mnih, V. *et al.* Asynchronous methods for deep reinforcement learning. In *International conference on machine learning*, 1928–1937 (2016).
9. Schulman, J., Moritz, P., Levine, S., Jordan, M. & Abbeel, P. High-dimensional continuous control using generalized advantage estimation. *arXiv preprint arXiv:1506.02438* (2015).
10. Gu, S., Lillicrap, T., Ghahramani, Z., Turner, R. E. & Levine, S. Q-prop: Sample-efficient policy gradient with an off-policy critic. *arXiv preprint arXiv:1611.02247* (2016).
11. Lillicrap, T. P. *et al.* Continuous control with deep reinforcement learning. *arXiv preprint arXiv:1509.02971* (2015).

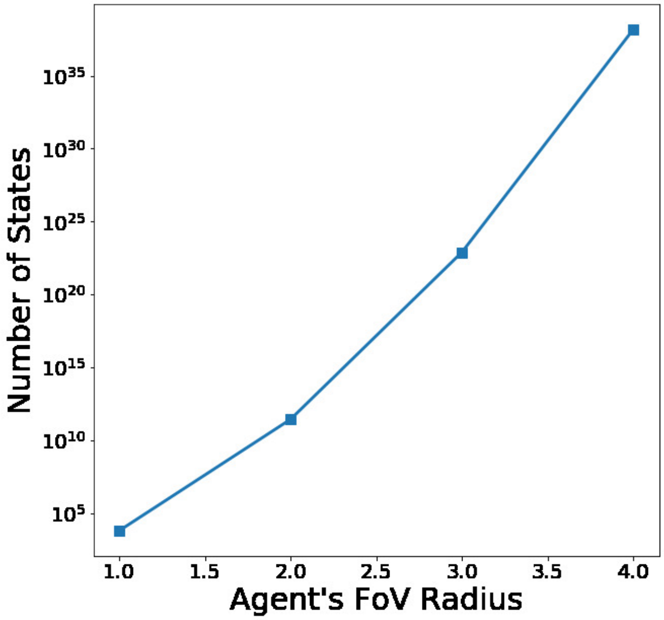

Figure S1. Number of states depending the radius r of agents Field of View (FOV) or observation window. Area of an agent's FOV is equal to n^2 where $n = 2r + 1$.

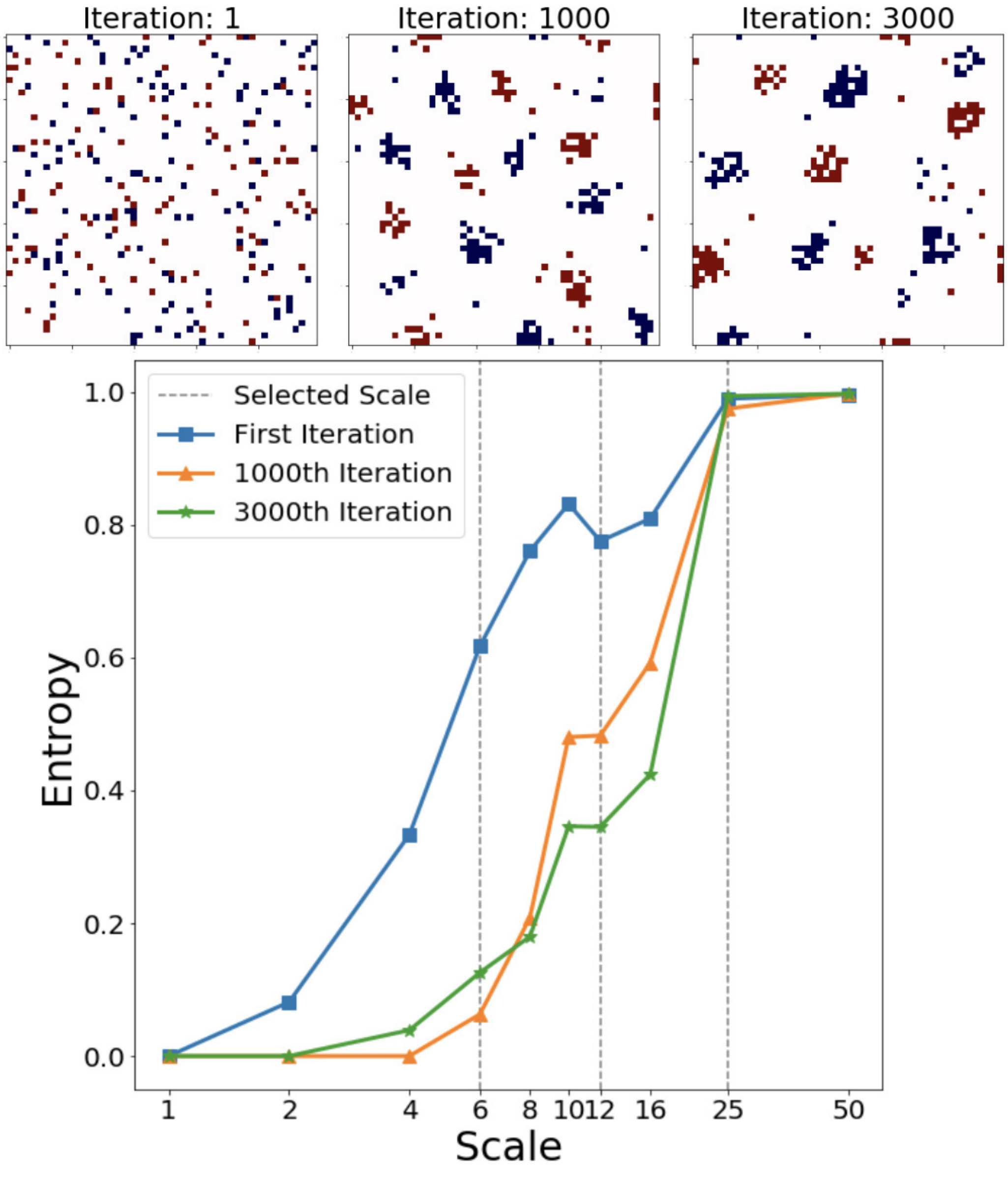

Figure S2. Spatial configurations of agents at various iterations (top). Corresponding entropy values across different scales (bottom). Dashed gray lines indicate the scales used in measuring multiscale entropy during experiments.

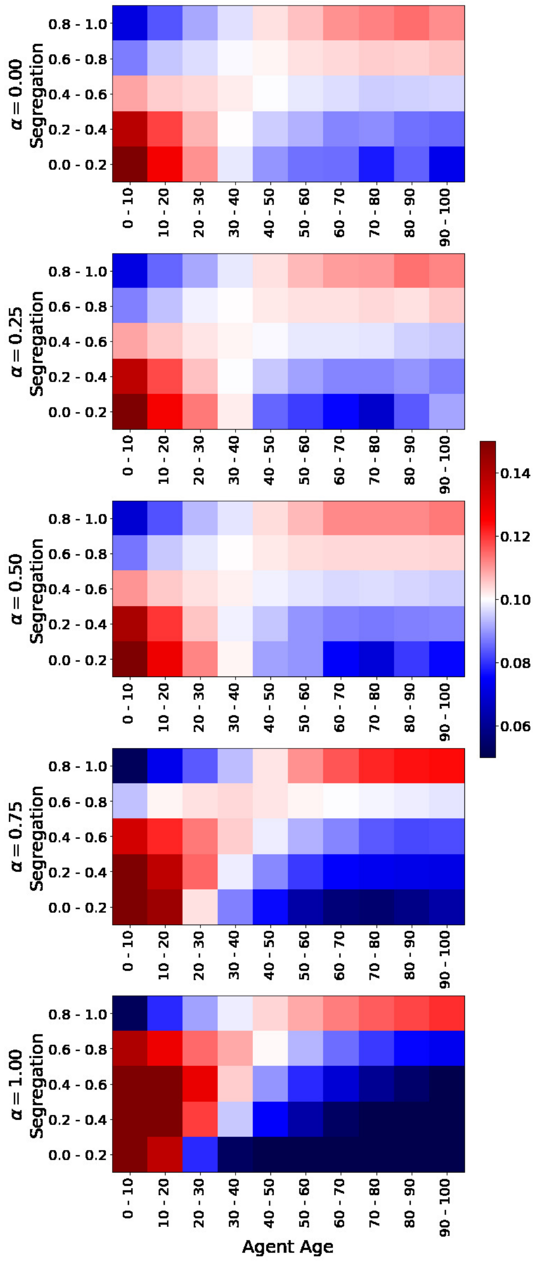

Figure S3. Probability distribution of age groups conditional on segregation of observation windows. Each panel shows the probabilities of finding agents at each age group (columns) at different levels of segregation in their observation windows (rows) during the last 1000 iterations. There is one panel per each value of the segregation parameter α. The interdependence reward IR=0 for all panels. Scale in Figure.

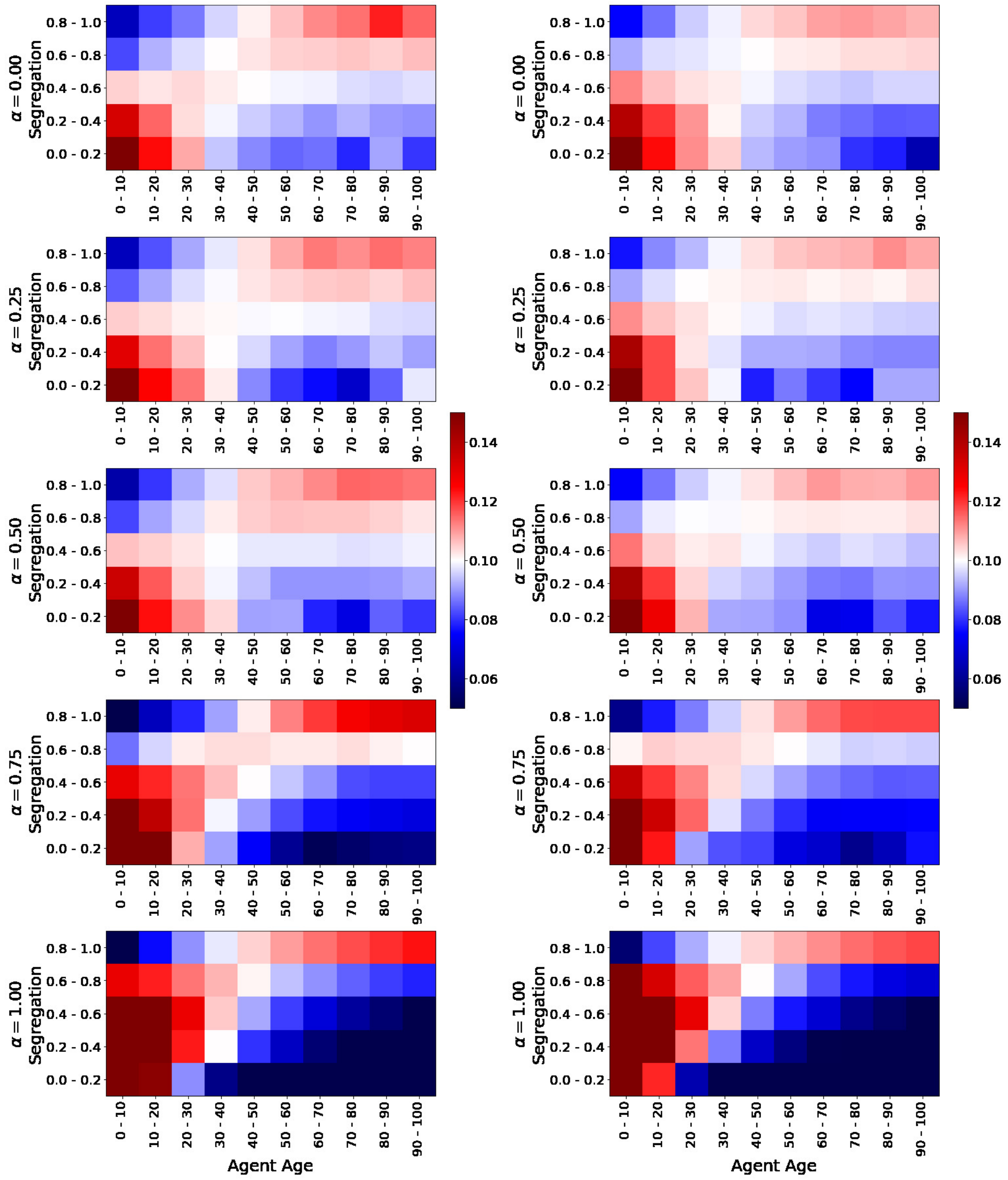

Figure S4. Probability distribution of age groups conditional on segregation of observation windows for agents A (left column) and B (right column). Each panel shows the probabilities of finding agents at each age group (columns) for each of the possible segregation value (rows) during the last 1000 iterations. There is one panel per each value of the segregation parameter α. The interdependence reward IR=0 for all panels. Scale in Figure.

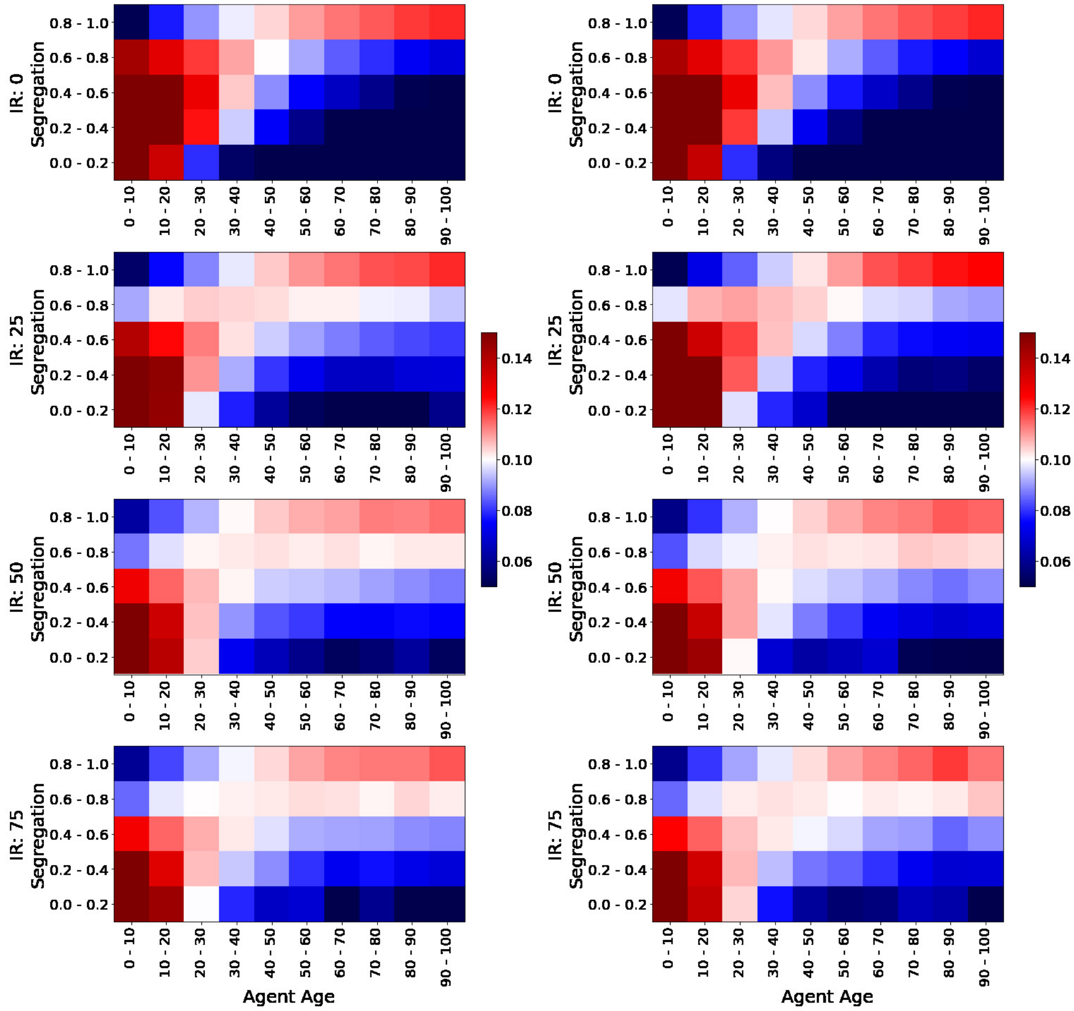

Figure S5. Probability distribution of age groups conditional on conditional on segregation of observation window for agents A (left column) and B (right column). Each panel shows the probabilities of finding agents at each age group (columns) for each of the possible actions (rows) during the last 1000 iterations. There is one panel per each value of interdependence reward (IR). The segregation parameter $\alpha = 1$ for all panels. Scale in Figure.

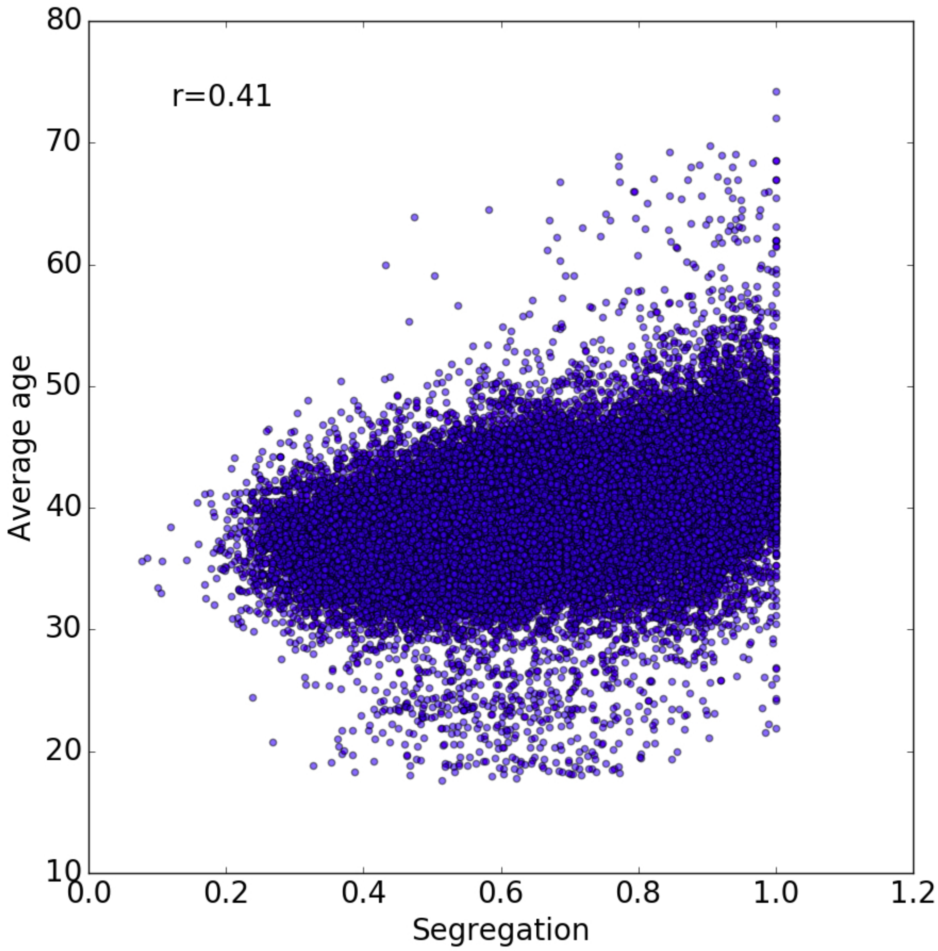

Figure S6. Age and racial segregation by census tract (dots). Pearson correlation r annotated in the Figure.

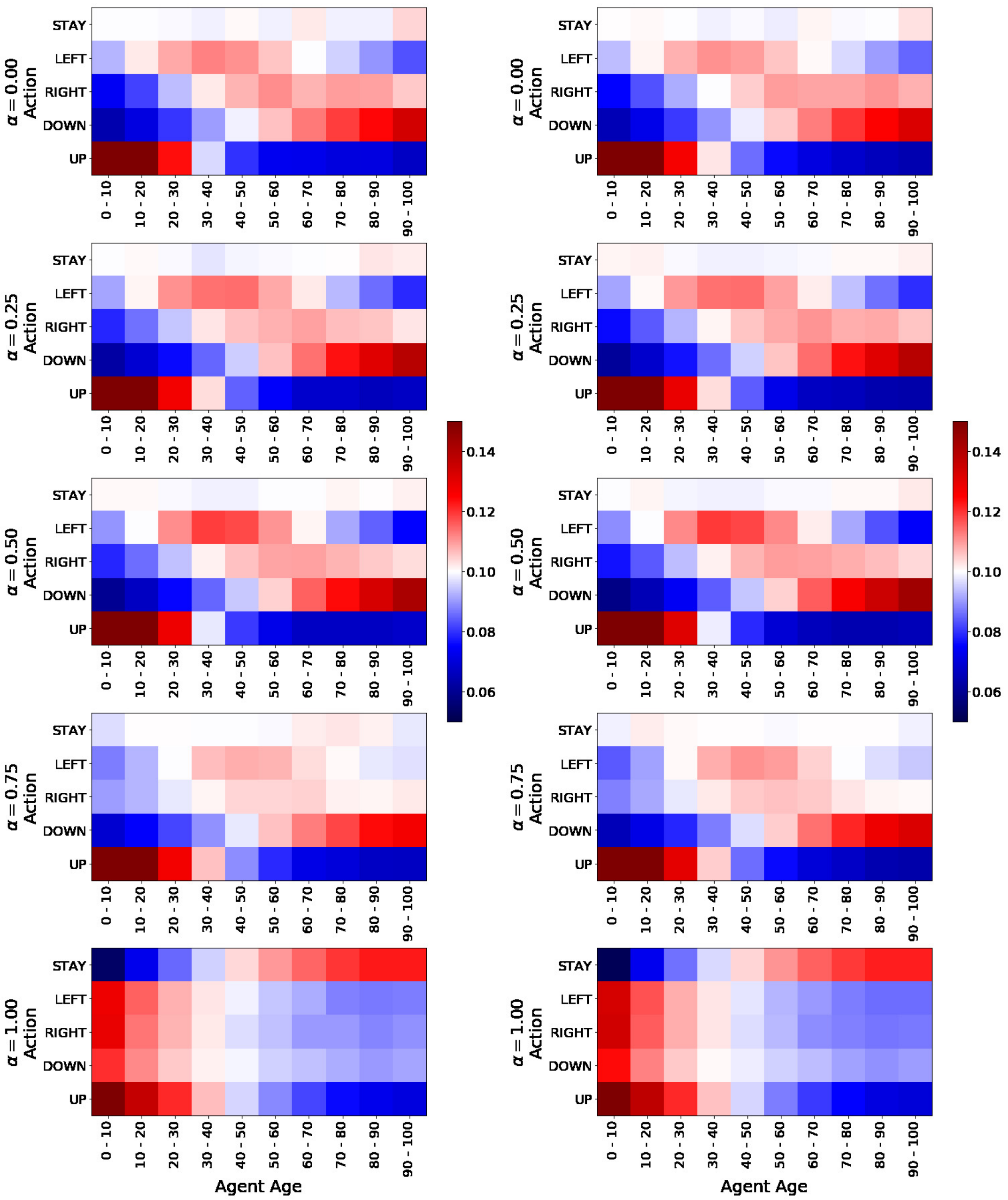

Figure S7. Probability distribution of age groups conditional on actions for agents A (left column) and B (right column). Each panel shows the probabilities of finding agents at each age group (columns) for each of the possible actions (rows) during the last 1000 iterations. There is one panel per each value of the segregation parameter α. The interdependence reward IR=0 for all panels. Scale in Figure.

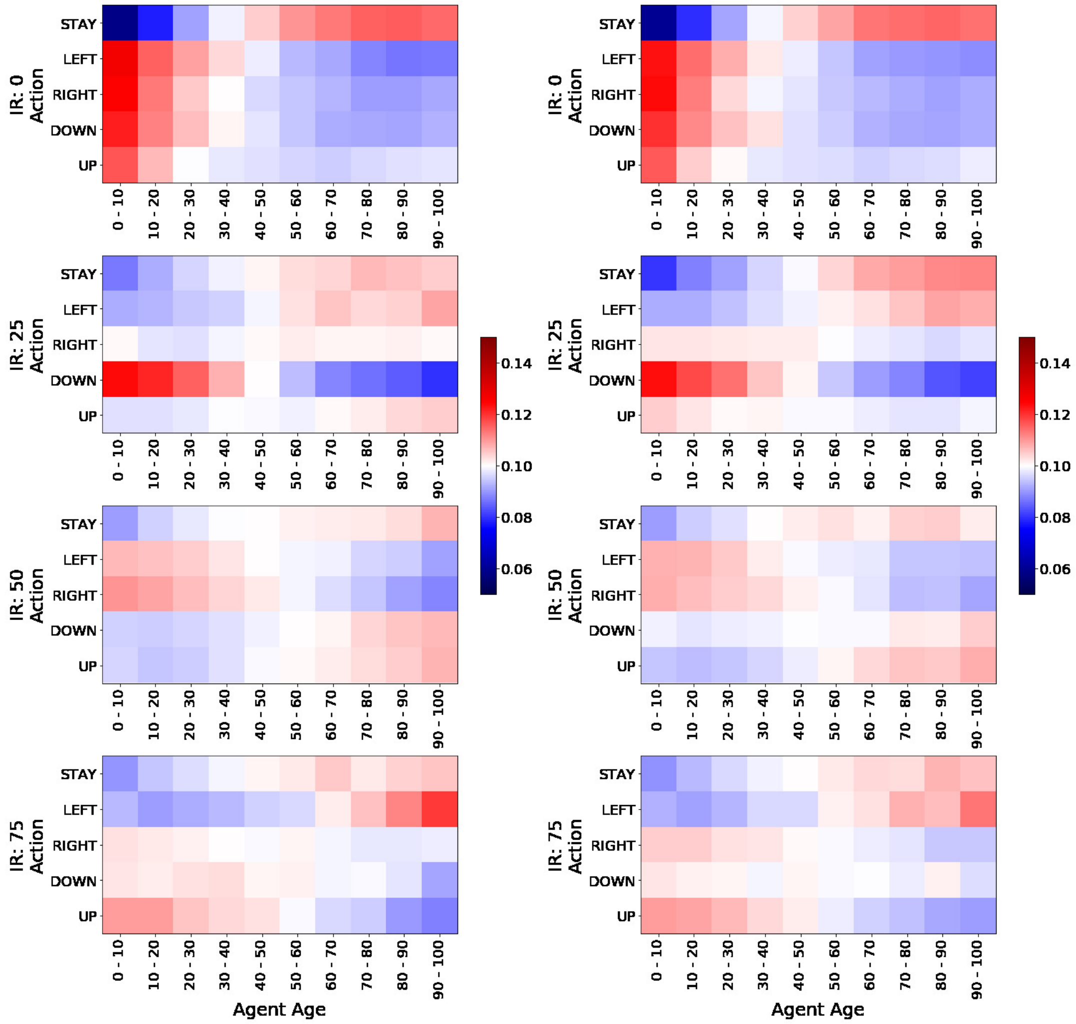

Figure S8. Probability distribution of age groups conditional on actions for agents A (left column) and B (right column). Each panel shows the probabilities of finding agents at each age group (columns) for each of the possible actions (rows) during the last 1000 iterations. There is one panel per each value of interdependence reward (IR). The segregation parameter $\alpha = 1$ for all panels. Scale in Figure.
